# Bat-Fruit Interactions Are More Specialized in Shaded-Coffee Plantations than in Tropical Mountain Cloud Forest Fragments

**DOI:** 10.1371/journal.pone.0126084

**Published:** 2015-05-18

**Authors:** Jesús R. Hernández-Montero, Romeo A. Saldaña-Vázquez, Jorge Galindo-González, Vinicio J. Sosa

**Affiliations:** 1 Instituto de Biotecnología y Ecología Aplicada (INBIOTECA), Universidad Veracruzana, Av. de las Culturas Veracruzanas No. 101, Col. E. Zapata, CP 91090, Xalapa, Veracruz, México; 2 Laboratorio de Ecología de Paisajes Fragmentados, Instituto de Investigaciones en Ecosistemas y Sustentabilidad, Universidad Nacional Autónoma de México, Antigua carretera a Pátzcuaro No. 8701, Ex-Hacienda de San José de la Huerta, 58190, Morelia, Michoacán, México; 3 Red de Ecología Funcional, Instituto de Ecología A.C. Apdo, Postal 63, CP 91000, Xalapa, Veracruz, México; Università degli Studi di Napoli Federico II, ITALY

## Abstract

Forest disturbance causes specialization of plant-frugivore networks and jeopardizes mutualistic interactions through reduction of ecological redundancy. To evaluate how simplification of a forest into an agroecosystem affects plant-disperser mutualistic interactions, we compared bat-fruit interaction indexes of specialization in tropical montane cloud forest fragments (TMCF) and shaded-coffee plantations (SCP). Bat-fruit interactions were surveyed by collection of bat fecal samples. Bat-fruit interactions were more specialized in SCP (mean *H_2_ ' *= 0.55) compared to TMCF fragments (mean *H_2_ '* = 0.27), and were negatively correlated to bat abundance in SCP (R = -0.35). The number of shared plant species was higher in the TMCF fragments (mean = 1) compared to the SCP (mean = 0.51) and this was positively correlated to the abundance of frugivorous bats (*R*= 0.79). The higher specialization in SCP could be explained by lower bat abundance and lower diet overlap among bats. Coffee farmers and conservation policy makers must increase the proportion of land assigned to TMCF within agroecosystem landscapes in order to conserve frugivorous bats and their invaluable seed dispersal service.

## Introduction

Seed dispersal by animals constitutes an important ecosystem service in natural forests and agroecosystems [1]. Study of the factors that modify animal-fruit interaction patterns is therefore important to the conservation of ecosystem functionality [2]. In ecology, network theory allows us to understand mutualistic interactions using different indices that describe the structure of interactions [3]. In seed dispersal networks, the specialization (*H*_*2*_ *'*) index is one of the main informative network indices, because is related to the complementarity of mutualistic interactions and thus describes the stability of the network by interaction redundancy [4].

Previous studies have found that specialization (*H*_*2*_ *'*) is negatively correlated to plant richness, canopy cover and forest disturbance. This is because a reduction in plant richness and canopy forest strata reduces the availability of fruit resources, causing the impoverishment of frugivorous diversity and animal-fruit interactions [5,6]. On the other hand, there is a positive relationship between specialization (*H*_*2*_ *'*) and fruit abundance, since the dominance of specific resources promotes the presence of specific frugivorous animals [6]. This information comes from bird-fruit interaction studies [5,6] but it is not known whether specialization of other animal-fruit interactions networks behave in a similar manner to bird-fruit interactions. This is particularly true when we take into consideration the fact that frugivorous birds have low diet overlap with other frugivorous animals, such as bats [7,8].

Frugivorous bats are important seed dispersers in tropical ecosystems, especially in disturbed forest [9]. Neotropical frugivorous bats feed on specific plant taxa; for example, bats of the *Carollia* and *Sturnira* genera feed mainly on understory fruits of the genera *Piper* and *Solanum*, respectively, while bats of the genus *Artibeus* feed mainly on canopy fruits of the genera *Ficus* and *Cecropia* [10,11]. Agricultural activities cause a reduction in the abundance of these chiropterochoric plants and in the abundance of bats specialized in their consumption [12]. This situation could affect bat-fruit interactions because a negative relationship exists between species abundance and specialization of interactions [13,14].

The mountainous central region of Veracruz, Mexico, offers an opportunity to test the effect of agricultural activities on the specialization of bat-fruit interaction networks. This region was originally occupied by tropical montane cloud forest (TMCF) located within an altitudinal strip at between 700–1800 masl. Landform and climate dictate the extensive cultivation of high-grown coffee between 700–1,400 masl [15], which causes the remaining TMCF fragments to have a higher density of chiropterochoric plants and frugivorous bats compared to shaded-coffee plantations (SCP) [16].

Shaded-coffee plantations are the main agroecosystem in the mountainous region of central Veracruz, Mexico. Due to the presence of an arboreal stratum, this agroecosystem harbors an important proportion of the regional biodiversity [17,18]. However, given the removal of the understory in SCP, chiropterochoric plants are less abundant than is the case in the TMCF [16]. In this study, we compare the specialization of bat-fruit interactions in TMCF and SCP. In addition, we evaluate the relationship between bat abundance and specialization in both vegetation types. We expect a higher specialization in SCP compared to TMCF fragments, due to the low abundance of understory chiropterochoric plants in SCP. In addition, we expect a negative relationship between bat abundance and specialization (*H*_*2*_ *'*) in SCP, because of the low abundance of frugivorous bats that produce only occasional bat-fruit interactions in SCP.

## Material and Methods

### Study area

The study was carried out in the municipalities of Xalapa and San Andrés Tlalnelhuayocan in central Veracruz, Mexico (19°31’19” to 19°29’46” N, 96°59’30” to 96°54’36” W), in two landscapes selected by a geographic information system created in ArcView 3.2. For details, see the map of study sites in [16]. The TMCF landscape (1268.11 ha) was dominated by a cattle ranching matrix (879.8 ha; 69%) with remnant TMCF fragments (388.31 ha; 31%). No SCP exists in this landscape. The SCP landscape (1253.91 ha) was dominated by a heterogeneous matrix comprising secondary TMCF fragments (265.86 ha; 21.2%), pastures and human settlements (645.77 ha; 51.5%). In this landscape, SCP comprised 342.29 ha (27.3%).

Forest cover in both landscapes was calculated using supervised classification from a LANDSAT-7 (2000) satellite image, while SCP cover was calculated using the agricultural census conducted by the Secretaría de Agricultura, Ganadería, Desarrollo Rural y Pesca (2000). We selected four TMCF fragments each in the TMCF and SCP landscapes. We also used the following selection criteria to select bat/seed sampling sites: (1) all forests patches and coffee plantations were chosen within the narrowest possible elevation range (1,300–1,500 m a.s.l.) in order to reduce the uncontrolled effect of altitude and mesoclimate; and (2) TMCF patches and SCP were located at least 7 km apart in order to avoid capturing the same individual bats in different landscapes on the same sample night. TMCF fragments or SCP replicates in each landscape window were located at least 1 km apart.

### Chiropterochoric plant species survey

We conducted a survey of chiropterochoric plant species within TMCF fragments and SCP in order to generate a reference collection of the bat-fruit and seeds of the region and assess the potential fruit resource availability in these vegetation types (see Table 1). In addition, this information allowed us to determine whether the bats consume fruits that grow in the plantations or only those from outside the plantations, since the surroundings of the SCP comprised other SCP, secondary forest fragments, and pastures that could contain chiropterochoric plant species.

**Table 1:** Number of bat-fruit interactions per vegetation type: Tropical montane cloud forest fragment (TMCF) or shaded-coffee plantation (SCP); and number of individuals of chiropterochoric plant species recorded in plant surveys.

	Bat-fruit interactions		Bat plant survey	
Plant species	TMCF	SCP	TMCF	SCP
Cecropiaceae				
Aff. *Cecropia*	1	2		
Chlorantaceae				
*Hedyosmum mexicanum*	60	1		
Clusiaceae				
Vismia mexicana*		20		
Melastomataceae				
*Miconia glaberrima^*	1			
Miconia mexicana*		1		
Moraceae				
*Ficus* (Urostigma)*		1		
Piperaceae				
*Piper lapathifolium*	85	7	106	3
*Piper hispidum*	61	3	37	1
*Piper auritum*	17	12	55	
Piperaceae spp 1^	2			
Piperaceae spp 2*		1		
Piperaceae spp 3^	1			
Piperaceae spp 4^	2			
Piperaceae spp 5*		1		
Rosaceae				
Eriobotrya japonica*		1		
Solanaceae				
*Solanum aphyodendron*	53	19		
*Solanum schlechtendalianum*	44	5	9	
*Solanum acerifolium*	2	4		
*Solanum diflorum^*	2			
*Lycianthes geminifolia*	6	12		
Solanaceae spp 1^	3			
Solanaceae spp 2	1	1		
Solanaceae spp 3^	4			
Solanaceae spp 4^	7			
Ulmaceae				
*Trema micrantha^*	12	1	2	4
Unidentified				
spp. 1^	5			
spp. 2*		1		
spp. 3*		1		
spp. 4^	1			
spp. 5^	1			
Total of bat-fruit interactions per vegetation type	371	94		
Total individual plants			209	8
Plant richness	22	19	5	3

^^^ denotes a plant species exclusive to the forest fragments;

* denotes a plant species exclusive to the coffee plantations.

We recorded the presence of chiropterochoric species along ten strip-transects (50 x 2 m) that were randomly established within each TMCF fragment or SCP, making a total of 0.1 ha of sampling area per site. The plant species survey was based on the database of Neotropical bat/plant interactions [19]. All trees or shrubs of height ≥1 m rooted within transects were recorded and identified. We collected specimens of those plants that could not be identified in the field, for subsequent determination in the XAL herbarium of the Instituto de Ecología, A.C. in Xalapa, Veracruz. All surveys were conducted during the bat sampling seasons (see below).

### Bat-fruit interaction data collection

Data on interactions between bats and fruit were obtained through fecal samples. Collection of fecal samples is a reliable method by which to assess frugivorous bat diet whenever samples contain identifiable seed or fruit pulp [20]. One fecal sample could have seeds from more than one plant species [21], but we counted seed deposition per plant species as a bat-fruit interaction. Thus, the number of fecal samples may not necessarily match with the number of bat-fruit interactions.

Sampling was carried out over the course of one year (June 2007-April 2008), covering the three climatic seasons recognized for the region: dry, wet, and the season of northerly cold fronts (“nortes”) [22]. We thus had 12 interaction matrices per vegetation type (four sites, sampled over three seasons). Bats were captured using eight mist nets (9 x 2.4 m, with a 14 x 14 mm mesh size) placed on the ground in each forest fragment and coffee plantation site for six nights, giving 24 nights of sampling per vegetation type. Mist nets were placed at ground level only, since there is no significant difference between canopy and ground mist nets in terms of the detectability of canopy frugivorous bats [23].

Mist nets were set in pairs some 15 to 30 m apart, within each fragment or plantation. Nets were opened at dusk for five hours and checked every 30 minutes. This produced a bat sampling effort of 20,736 m^2^·h [24] in each vegetation type. To reduce the effects of variable weather, successive sampling nights were alternated between vegetation types. Dates around the full moon were avoided, since frugivorous bat activity declines during this period [25]. In forests, bats move through understory areas with little foliage, which facilitates their movement [26]; mist nets were therefore placed diagonally across man-made trails. In the SCP, mist nets were placed along the lanes that delimit the plantation sections. Captured bats were identified using field keys [27], tagged with a numbered plastic collar and released at the site of capture.

We used two complementary methods to collect fecal samples. First, we placed a plastic sheet (9 x 1 m) below each net in order to gather droppings deposited before the bat was removed from the net [21]. In addition, the canvas bags used for holding bats (< 30 min) were also inspected for feces. To avoid possible confusion in the assignation of the fecal sample to the captured bat we visited the mist-nets every 30 min. On rare occasions, two bats became entangled in the net simultaneously; however, we were always able to identify which individual produced the scats by looking for seeds remaining on the bat and net, by the color or type of seeds on the plastic sheet and/or by the vertical position of the droppings under the bat.

Plant seeds defecated by bats were identified to the lowest possible taxonomic level (family, genus or species) by comparison to our reference collection using a stereoscopic microscope (20×). Seeds of fecal samples that did not match with the reference collection were classified as morphospecies.

### Data analysis

We evaluated the completeness of bat-fruit interactions sampling by vegetation type and compared the richness of seed consumed by bats between vegetation types. In the former case, we compared the observed seed richness to the expected richness in each vegetation type. In the latter case, we compared the observed seed richness of the two vegetation types. Since we did not count the number of seeds of plant species in each fecal sample, we selected the bootstrap estimator of richness, which is sensitive to species incidence. Individual bats that produced fecal samples were considered as the sample unit for this analysis [28]. Values for the observed species richness, as well as the bootstrap-estimated species richness with their respective confidence intervals, were obtained by rarefaction of all pooled samples with 100 randomizations without replacement using EstimateS [29]. To determine significant difference in dispersed seed richness between vegetation types, we compared the 84% confidence intervals (CI) that robustly mimic the 0.05 statistical test for asymmetric CI at α ≤ 0.05 [30]. Where the 84% CI overlapped, we considered that seed dispersed richness did not differ statistically.

In order to evaluate spatial autocorrelation and pseudo-replication among sampling/replication sites, we conducted a Mantel test [31]. Originally, this analysis compares the observed correlation between genetic similarity and geographic distance versus the distribution of correlation values of randomized matrices. In our case, the observed correlation was between plant seed richness, number of fecal samples and seed composition similarity among sites of each vegetation type and geographic distance among sampling sites. The Euclidean method was used for the calculation of seed richness, similarity of number of fecal samples and geographic distances matrices, while the Bray-Curtis method was selected for calculating seed composition similarity. The number of randomizations of the matrices values was 9,999.

To compare bat-fruit interactions specialization between vegetation types and their possible interaction with climatic season, we constructed interaction matrices by site and season (see the interaction matrices in S1 Appendix). Two indexes related to specialization of ecological networks were calculated: specialization index (*H*_*2*_ *'*), and mean number of shared species consumed by bats of the network. The specialization index (*H*_*2*_ *'*) describes the complementarity of interactions among members of a network. Values of *H*_*2*_ *'* range from zero to one; values approaching zero suggest a high complementarity of interactions (low specialization) or high redundancy of interactions in the network, while values approaching one suggest low complementarity (high specialization) of interactions of the network [32]. Compared to highly specialized networks, those with low values of *H*_*2*_ *'* can more easily compensate and maintain their stability in the event of a disturbance or fluctuation in environmental conditions [3]. The underlying equation is the same as the two-dimensional Shannon entropy, however, the value calculated for the given network (*H*_*2*_) is standardized against the minimum (*H*_*2*min_) and maximum (*H*_*2*max_) possible values for the same distribution of interaction:

$$H_{2}\prime =\frac{(H_{2\max}-H_{2}}{\left(H_{2\max}-H_{2}\min\right)}$$

The mean number of shared species index reveals the mean number of plants consumed by a pair of bat species in the interaction matrix data [33]. To compare the *H*_*2*_ *'* and the mean number of shared species values between TMCF fragments and SCP, we used generalized linear models (GLM). A GLM with a post-hoc χ^2^ analysis for standardized coefficients and gamma distribution of errors was used for the *H*_*2*_ *'* index because the index values do not follow a normal distribution. For the mean number of shared species index values, we used a normal error distribution (equivalent to a standard ANOVA). The syntax for fitting each linear model was as follows: glm/aov (‘index value’ ~ habitat * season, ‘error’).

We evaluated the dependency of specialization indexes on frugivorous bat abundance using analysis of covariance (ANCOVA) procedures. Included in this analysis were the specialization indexes calculated by each site in turn as the dependent variable, the frugivorous bat abundance of each site as the continuous variable, and vegetation type as a factor. The addition of vegetation type as a factor allows us to detect possible differences among slopes (significant effect of the bat abundance-vegetation interaction) or intercepts (significant effect of the vegetation factor). Since the specialization index residuals were not normally distributed and the relationship between any index and bat abundance was non-linear, we transformed both the indexes and the abundance values to log(x+1). The frugivorous bat abundance data came from all disperser bats species captured at each site, including bats that produced no fecal samples. All analyses were performed using the vegan, ade4, bipartite and stats libraries of the R 2.12.2 software [34–36].

## Results

**Fig 1:**
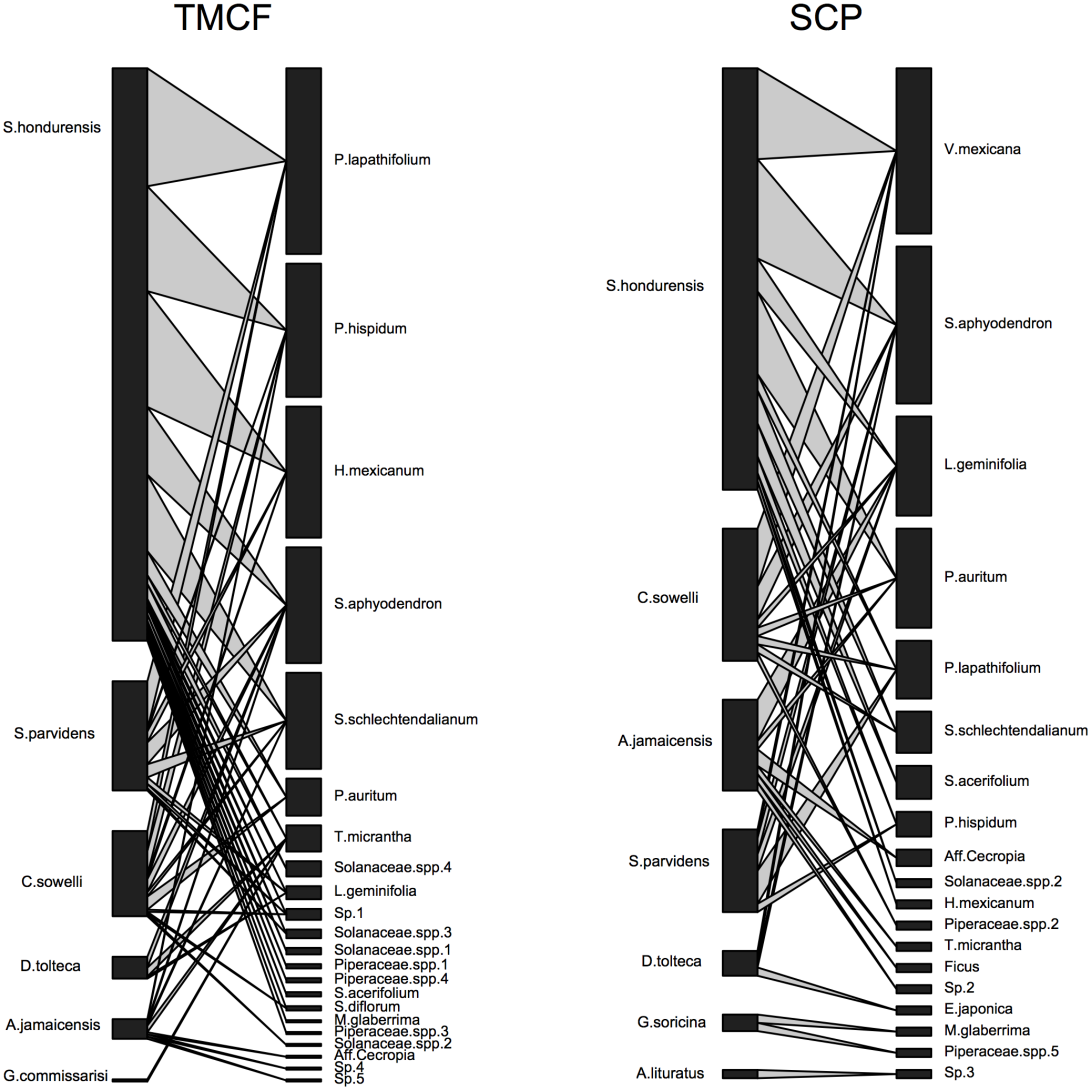
Quantitative bipartite plant-bat interaction graph for tropical montane cloud forest fragments (TMCF) and shaded-coffee plantations (SCP). For each bipartite graph, the right-hand bar size represents the number of plant species in fecal samples and left-hand bar size represents the number of bats for which a fecal sample was obtained. Linkage width indicates the frequency of each trophic interaction, in TMCF were recorded 371 interactions, while in SCP 94.

In total, we recorded 768 bats belonging to 16 species in three families (see S1 Table). Eight frugivorous bat species were recorded; six in TMCF fragments and seven in SCP (Fig 1). We recorded fruit consumption in nectarivorous bats of the genus *Glossophaga*; this genus includes species that are able to consume seasonal fruits in the absence of nectar resources [37]. We observed a total of 465 fecal samples (371 in TMCF *vs*. 94 in SCP), with seeds of 30 plant species from both vegetation types (Table 1). In the TMCF, 259 fecal samples had seeds of one plant species, 48 had seeds of two plant species, four had seeds of three plant species and one fecal sample contained the seeds of four different plants species. In the SCP, 72 fecal samples had seeds of one plant species and 11 fecal samples had seeds of two plant species. All recaptures occurred in replicates of the same vegetation type, i.e. we did not recapture any individuals in a vegetation type that differed from that in which they were first captured.

We found no autocorrelation between ecological and geographic distance of our sampling sites (TMCF richness: *r* = 0.49, *P* = 0.17; SCP richness: *r* = 0.30, *P* = 0.24; TMCF fecal samples: *r* = 0.52, *P* = 0.12; SCP fecal samples: *r* = –0.11, *P* = 0.61; and TMCF seed composition: *r* = 0.50, *P* = 0.16; SCP seed composition: *r* = –0.07, *P* = 0.50). These results indicate that our bat-plant sampling sites provided spatially independent data. Our chiropterochoric plant species survey within the SCP revealed a lower density of chiropterochoric plants compared to that of the TMCF fragments (Table 1). Evaluation of the bat-fruit interaction survey showed no differences between the observed and estimated seed richness in the TMCF fragments, and SCP (Table 2). There were no differences in the observed richness of dispersed seeds between the TMCF fragments and the SCP (Table 2).

**Table 2:** Observed and estimated seed richness by the bootstrap predictor in tropical montane cloud forest fragments (TMCF), and shaded-coffee plantation (SCP).

	TMCF	SCP
Observed richness	22 ± 3	19 ± 5
Estimated richness	25 ± 0	23 ± 1

Error values represent a confidence interval of ± 84%.

Bat-fruit interactions networks were more specialized in the SCP than in the TMCF fragments (GLM: χ^2^ = 0.29, df = 1, *P* = 0.03, Table 3) and there was neither a seasonal effect nor an interaction between season and vegetation (season: χ^2^ = 0.18, df = 2, *P* = 0.15; season-vegetation interaction: χ^2^ = 0.20, df = 2, *P* = 0.13). The mean number of species shared by bats was higher in the TMCF fragments than in SCP (*F* = 10.61, df = 1, *P* = 0.005, Table 3) and again there was neither a seasonal effect nor an interaction between season and vegetation (season: *F* = 3.18, df = 1, *P* = 0.07; season-vegetation interaction: *F* = 1.04, df = 1, *P* = 0.37).

**Table 3:** Specialization index comparisons of bat-fruit interactions in tropical montane cloud forests fragments (TMCF) and shaded-coffee plantations (SCP).

	TMCF	SCP
Mean shared plants	1 ± 0.06	0.5 ± 0.16
Specialization (*H*_*2*_ *'*)	0.27 ± 0.05	0.55 ± 0.15

Values represent the mean and one standard error.

**Fig 2:**
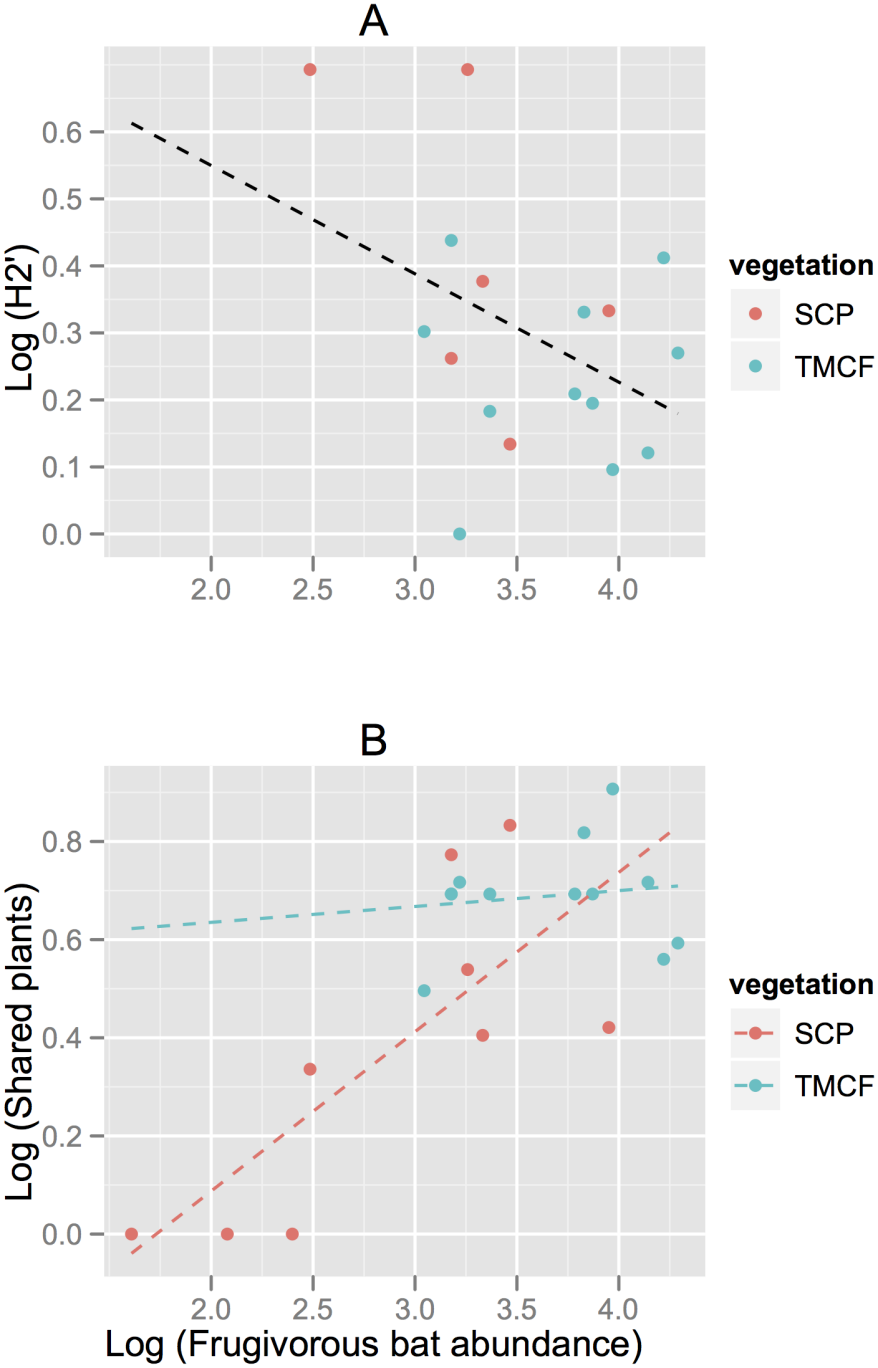
Relationship between frugivorous bat abundance and specialization indices in tropical montane cloud forests fragments (TMCF) and shaded-coffee plantations (SCP). A: specialization (*H*_*2*_ *'*); B: mean shared plants species. Dotted lines represent predicted linear models.

Since it was not justified to assume differences in the linear relationship between vegetation types, SCP *vs*. TMCF (*t* = -1.673, *P* = 0.12 for intercepts; *t* = 1.482, *P* = 0.16 for slopes), we simplified the ANCOVA model by retiring the effect of vegetation. After simplifying the model, we found a negative relationship between frugivorous bat abundance and *H*_*2*_ *'* (*R* = -0.35), although this was not statistically significant (*F* = 3.19; df = 1, 15; *P* = 0.09; Fig 2A). On the other hand, there was a significant positive linear relationship between bat abundance and mean number of plant species shared by bats (*F* = 30.1; df = 17; *P* = 0.0004); and a difference in the slopes (*t* = -2.17, *P* = 0.04) and intercepts (*t* = -2.42, *P* = 0.027) of the lines fitted to each vegetation type (TMCF *b* = 0.57; SCP *b* = 0.01; *t* = -2.16; *P* < 0.05; Fig 2B).

## Discussion

We hypothesized that specialization of bat-fruit interactions is affected by the lower chiropterochoric plant and bat abundance in SCP. The higher specialization of SCP bat-fruit interaction networks and the negative relationship between bat abundance and *H*_*2*_ *'* support the hypothesis that specialization is related to bat abundance. In the following sections, we discuss the possible factors that may affect specialization of ecological networks in agroecosystems such as SCP and the consequences for bat-fruit interaction networks. Finally, we discuss the implications of our results for the conservation of frugivorous bats and the valuable seed dispersal service they perform.

### Specialization in bat-fruit networks in agroecosystems

The lower values of specialization in TCMF fragments could be due to the high density of chiropterochoric plants and bats in this vegetation type compared to SCP [16]. Low *H*_*2*_ *'* values in TMCF reflect the high complementarity of the bat-fruit interactions (see S1 Appendix). In contrast, the low density of bats in SCP results in a high specialization of networks and less shared plants dispersed by bats (see Table 3 and Fig 2B). These results are consistent with previous studies showing a negative relationship between species abundance and specialization of interactions [13,14].

The values of the specialization index observed in SCP (mean *H*_*2*_ *'* = 0.55) and TCMF fragments (mean *H*_*2*_ *'* = 0.27) are in the range reported previously for Neotropical bat-fruit interactions (*H*_*2*_ *'* = 0.18–0.51). The fluctuation in specialization values is not related to the richness of plants or bats [38]. For example, bat-plant interactions networks with seven species of bats and 12 seed dispersed plants species had *H*_*2*_ *'* value of 0.51, while networks with 14 species of bats and 36 seed dispersed plants species had *H*_*2*_ *'* value of 0.31 [38]. This suggests that the changes in specialization values are better explained by the diet overlap and abundance of frugivorous bats rather than the richness of bats or seed dispersed plants. This hypothesis is supported by the similar bat and chiropterochoric plant richness found in TMCF and SCP and by their significantly different values of *H*_*2*_ *'*.

Another interesting result of our study is the large number of plant species detected in fecal samples that were not observed in our plant surveys. While it is possible that our chiropterochoric plant survey may be incomplete, we consider that this does not affect the principal result of the study since the observed richness of dispersed seeds did not differ significantly from the estimated richness (see Table 2). Previous studies have recorded seed dispersed plants that were not observed in our plant survey in primary TMCF, secondary TMCF and SCP [39,40]. This suggests that bats consumed plants in vegetation types similar to those we studied and that the chiropterochoric flora of each plantation is only a reduced portion of the chiropterochoric flora of the fragmented landscape. This is specially true for canopy plants as *Hedyosmum mexicanum*, *Vismia mexicana*, and Solanaceae understory plants (see Table 1).

The higher specialization of interactions and the lower bat abundance in SCP could have important consequences for seed dispersal and forest regeneration in coffee landscapes. One consequence could be that the seed dispersal service provided by bats could be less stable in the SCP. For example, bats in SCP on average shared less than one plant (Table 3). If we take into account that networks with high complementarity of interactions can compensate and maintain their stability when a disturbance or fluctuating environmental condition appears [3,32], frugivorous bats in SCP could have a lower probability of maintaining a seed dispersal service compared to those in the TMCF fragments.

### Bat-fruit specialization and the conservation of frugivorous bats

SCP could reduce the abundance of frugivorous bats and increase the commuting distance to foraging sites [16,41]. The low abundance of frugivorous bats result in high specialization in bat-fruit interactions and the vulnerability of the interaction network in SCP. Therefore, in order to assist in the conservation efforts of these seed-dispersing bats and to maintain forest regeneration potential in SCP coffee farmers and policy makers should: (1) attempt to increase the proportion of land assigned to forest within the agricultural landscape, and (2) use the measurement of the diversity and abundance of frugivorous bats as an indicator that SCP have sufficient food resources for frugivorous bats around and within the plantations. The first management recommendation is specially important if we take into account that a great number of understory plants consumed by bats came from forest.

In conclusion, in the SCP, bat-plant interaction networks had higher specialization values, bats shared a lower number of plant species and potentially dispersed a lower number of seeds of understory plants. These results are the consequence of low frugivorous bat abundance in the SCP and, under scenarios of coffee plantation expansion, represent trade-offs for the conservation of frugivorous bats and the invaluable seed dispersal service they provide.

## Supporting Information

S1 Appendix Bat-fruit interaction matrices by site and season. Letter B refers to TMCF, while letter C refers to SCP.
(PDF)

S1 Table Captured bat species in tropical mountain cloud forest fragments (TMCF) and shade coffee plantations (SCP). Guild bat classification is based on Rojas et al. 2012.
(PDF)
