# Supplementary material for: Bat-Fruit Interactions Are More Specialized in Shaded-Coffee Plantations than in Tropical Mountain Cloud Forest Fragments
Source: PLoS One. 2015 May 18;10(5):e0126084. doi: 10.1371/journal.pone.0126084 (PMC4436294; doi:10.1371/journal.pone.0126084)
Supplement: S1 Appendix — Letter B refers to TMCF, while letter C refers to SCP. (PDF) [file pone.0126084.s001.pdf]

S1 Appendix. Bat-fruit interaction matrices by site and season. Letter B refers to TMCF, while letter C refers to SCP.

B1 Wet	*Artibeus jamaicensis*	*Artibeus tolteca*	*Sturnira parvidens*	*Sturnira hondurensis*
Aff. *Cecropia*	1	0	0	0
Hedyosmum mexicanum	0	0	0	7
Piper hispidum	0	0	0	23
Piper lapathifolium	0	0	0	8
Piper spp 1	0	0	0	2
Solanum acerifolium	0	0	0	1
Solanum aphyodendron	0	1	0	10
Solanum schlechtendalianum	0	0	1	7

B1 Nortes	*Carollia sowelli*	*Sturnira hondurensis*
Hedyosmum mexicanum	1	19
Piper auritum	1	0

B1 Dry	*Carollia sowelli*	*Sturnira parvidens*	*Sturnira hondurensis*
Hedyosmum mexicanum	0	2	2
Piper hispidum	0	1	0
Piper lapathifolium	2	7	8
Piper spp 4	0	0	1
Solanaceae spp 4	0	0	6
Solanum aphyodendron	0	2	1
Solanum schlechtendalianum	0	0	3

B2 Wet	*Artibeus jamaicensis*	*Carollia sowelli*	*Sturnira hondurensis*
Hedyosmum mexicanum	0	0	2
Piper auritum	0	0	2
Piper hispidum	1	5	11
Piper lapathifolium	0	0	7
Solanaceae spp 1	0	0	1
Solanum aphyodendron	0	1	6
Solanum schlechtendalianum	0	0	3

B2 Nortes	*Carollia sowelli*	*Dermanura tolteca*	*Sturnira hondurensis*
Hedyosmum mexicanum	0	0	9
Miconia glaberrima	0	0	1
Piper auritum	2	0	2
Solanum aphyodendron	0	1	2
Solanum schlechtendalianum	0	0	1

B2 Dry	*Carollia sowelli*	*Sturnira parvidens*	*Sturnira hondurensis*
Hedyosmum mexicanum	1	3	4
Spp 1	0	2	1
Piper hispidum	1	0	0
Piper lapathifolium	3	6	9
Solanaceae spp 3	0	1	3
Solanum aphyodendron	2	6	1
Solanum schlechtendalianum	0	1	0

B3 Wet	*Carollia sowelli*	*Sturnira hondurensis*
Hedyosmum mexicanum	0	1
Piper hispidum	2	8
Piper lapathifolium	0	4
Piper spp 3	0	1
Solanum aphyodendron	0	2
Solanum schlechtendalianum	0	8

B3 Nortes	*Artibeus jamaicensis*	*Carollia sowelli*	*Sturnira parvidens*	*Sturnira hondurensis*
Hedyosmum mexicanum	0	0	0	2
Lycianthes geminifolia	0	0	0	1
Spp 5	1	0	0	0
Piper auritum	0	0	0	3
Solanaceae spp 2	0	1	0	0
Solanum acerifolium	0	0	0	1
Solanum diflorum	0	1	0	1
Solanum schlechtendalianum	0	1	2	5

B3 Dry	*Carollia sowelli*	*Sturnira parvidens*	*Sturnira hondurensis*
Lycianthes geminifolia	0	1	0
Piper hispidum	0	0	1
Piper lapathifolium	1	3	4
Piper spp 4	0	0	1
Solanaceae spp 4	0	0	1

Solanum aphyodendron	1	1	2
Solanum schlechtendalianum	0	1	1

B4 Wet	*Artibeus jamaicensis*	*Carollia sowelli*	*Sturnira parvidens*	*Sturnira hondurensis*
Spp 4	1	0	0	0
Piper auritum	0	0	0	1
Piper hispidum	0	2	0	5
Piper lapathifolium	0	1	0	4
Solanaceae spp1	0	0	0	2
Solanum aphyodendron	0	0	0	5
Solanum schlechtendalianum	1	0	1	3

B4 Nortes	*Artibeus jamaicensis*	*Carollia sowelli*	*Dermanura tolteca*	*Glossophaga commissarisi*	*Sturnira parvidens*	*Sturnira hondurensis*
Hedyosmum mexicanum	0	0	0	0	0	6
Lycianthes geminifolia	0	0	1	0	1	1
Piper auritum	0	3	0	0	0	3
Solanum aphyodendron	0	0	2	0	0	1
Solanum schlechtendalianum	0	1	0	0	0	4
Trema micrantha	3	0	4	1	0	4

B4 Dry	*Artibeus jamaicensis*	*Carollia sowelli*	*Dermanura tolteca*	*Sturnira parvidens*	*Sturnira hondurensis*
Hedyosmum mexicanum	0	0	0	0	1
Lycianthes geminifolia	0	0	0	1	0
Spp 1	0	1	0	0	1
Piper hispidum	0	1	0	0	0

Piper lapathifolium	0	2	0	6	10
Solanum aphyodendron	1	2	1	1	1

C1 Wet	*Carollia sowelli*	*Glossophaga soricina*
Miconia mexicana	0	1
Piper spp 2	1	0

C1 Nortes	*Artibeus jamaicensis*	*Carollia sowelli*	*Sturnira parvidens*	*Sturnira hondurensis*
Lycianthes geminifolia	0	1	0	1
Piper auritum	1	1	0	2
Solanum acerifolium	0	0	0	1
Solanum schlechtendalianum	0	0	0	2
Vismia mexicana	0	2	1	1

C1 Dry	*Artibeus jamaicensis*	*Carollia sowelli*	*Sturnira parvidens*	*Sturnira hondurensis*
Ficus (Urostigma)	1	0	0	0
Lycianthes geminifolia	1	0	1	0
Spp 2	1	0	0	0
Piper lapathifolium	0	1	0	2
Solanum aphyodendron	0	1	0	3

C2 Wet	*Artibeus jamaicensis*	*Sturnira hondurensis*
Aff. *Cecropia*	1	0
Piper auritum	0	1
Piper hispidum	0	1
Solanum aphyodendron	0	2
Trema micrantha	1	0

C2 Nortes	*Artibeus jamaicensis*	*Sturnira hondurensis*
Lycianthes geminifolia	1	0
Piper auritum	0	1
Solanaceae spp 2	0	1
Solanum acerifolium	0	1
Solanum aphyodendron	0	1
Solanum schlechtendalianum	0	2
Vismia mexicana	0	3

C2 Dry	*Artibeus jamaicensis*	*Carollia sowelli*	*Sturnira parvidens*	*Sturnira hondurensis*
Lycianthes geminifolia	2	0	1	2
Piper hispidum	0	0	1	0
Piper lapathifolium	0	0	3	0
Solanum aphyodendron	0	2	2	3

C3 Wet	*Sturnira hondurensis*
Lycianthes geminifolia	1
Piper auritum	1
Piper hispidum	1

C3 Nortes	*Artibeus jamaicensis*	*Carollia sowelli*	*Dermanura tolteca*
Eriobotrya japonica	0	0	1
Lycianthes geminifolia	1	0	0
Vismia mexicana	0	1	0

C3 Dry	*Sturnira parvidens*
Piper lapathifolium	1

C4 Wet	*Artibeus jamaicensis*	*Glossophaga soricina*
Aff. *Cecropia*	1	0
Piper spp 5	0	1

C4 Nortes	*Artibeus lituratus*	*Carollia sowelli*	*Dermanura tolteca*	*Sturnira hondurensis*
Hedyosmum mexicanum	0	0	0	1
Spp 3	1	0	0	0
Piper auritum	0	0	0	5
Solanum acerifolium	0	0	0	2
Solanum aphyodendron	0	0	0	3
Solanum schlechtendalianum	0	1	0	0
Vismia mexicana	0	4	1	7

C4 Dry	*Carollia sowelli*	*Dermanura tolteca*
Solanum aphyodendron	1	1
