# Supplementary material for: Bat-Fruit Interactions Are More Specialized in Shaded-Coffee Plantations than in Tropical Mountain Cloud Forest Fragments
Source: PLoS One. 2015 May 18;10(5):e0126084. doi: 10.1371/journal.pone.0126084 (PMC4436294; doi:10.1371/journal.pone.0126084)
Supplement: S1 Table — Guild bat classification is based on Rojas et al. 2012. (PDF) [file pone.0126084.s002.pdf]

S2 Table. Captured bat species in tropical mountain cloud forest fragments (TMCF) and shade coffee plantations (SCP). Guild bat classification is based on Rojas et al. 2012.

Species	Guild	TMCF	SCP	Total
**Stenodermatinae**				
Artibeus jamaicensis	Frugivore	41	48	89
Artibeus lituratus	Frugivore	2	11	13
Dermanura tolteca	Frugivore	23	9	32
Centurio senex	Frugivore	0	3	3
Sturnira hodurensis	Frugivore	329	95	424
Sturnira parvidens	Frugivore	63	12	75
**Carollinae**				
Carollia sowelli	Frugivore	47	17	64
**Glossophaginae**				
Anoura geoffroyi	Nectarivore	3	13	16
Glossophaga soricina	Nectarivore	0	6	6
Glossophaga commissarisi	Nectarivore	1	5	6
**Desmodontinae**				
Desmodus rotundus	Saguinivore	13	0	13
**Verpertelionidae**				
Myotis keaysi	Insectivore	6	2	8
**Mormoopidae**				
Mormoops megalophylla	Insectivore	15	2	17
Pteronotus davyi	Insectivore	0	1	1
Pteronotus parnelli	Insectivore	1	0	1
**Total**		544	224	768
**Richness (S)**		12	14	15

Rojas D, Vale Á, Ferrero V, Navarro L (2012) The role of frugivory in the diversification of bats in the Neotropics. Journal of Biogeography 39: 1948–1960. doi:10.1111/j.1365-2699.2012.02709.x.
